# Recent Advances in Nanoplatforms for the Treatment of Osteosarcoma

**DOI:** 10.3389/fonc.2022.805978

**Published:** 2022-02-15

**Authors:** Kunzhe Wu, Beibei Yu, Di Li, Yangyang Tian, Yan Liu, Jinlan Jiang

**Affiliations:** ^1^ Department of Scientific Research Center, China-Japan Union Hospital of Jilin University, Changchun, China; ^2^ Department of Anesthesiology, China-Japan Union Hospital of Jilin University, Changchun, China; ^3^ Department of Dermatology, China-Japan Union Hospital of Jilin University, Changchun, China

**Keywords:** nanoplatform, drug delivery system, osteosarcoma, tumor targeted therapy, nanoparticles

## Abstract

Osteosarcoma (OS) is the most common primary bone tumor in children and young people. Traditional surgical excision combined with chemotherapy presents many limitations, such as resistance and systemic side effects of chemotherapy drugs, postoperative recurrence, and bone defects. Given these limitations, novel therapeutic modalities for OS treatment using nanometer-sized platform-based chemotherapeutic delivery have emerged as a promising alternative therapy. This form of therapy offers multiple advantages, such as accurate delivery of the drug to the tumor site and repair of limited bone defects after tumor resection. In this review, we briefly summarize nanoplatforms, including liposomes, polymeric nanoparticles, inorganic nanoparticles, nanomicelles, dendrimers, nanocapsules, and exosomes. The essential shortcomings involved in these nanoplatforms, such as poor stability, immunogenicity, insufficient circulation, and drug leakage are also discussed, and related solutions are briefly proposed. Finally, the application prospects of nanoplatforms in the treatment of OS are discussed.

## 1 Introductions

Osteosarcoma (OS) is the most common primary malignant bone tumor among children and adolescents and often involves the metaphysis of the long diaphysis (1). Surgical resection combined with chemotherapy has increased the 5-year survival rate of patients with OS from 20% to 70%, but the survival rate of patients with advanced OS remains less than 30% (2). In addition, owing to chemotherapy drug resistance and poor selectivity, the effect of the treatment has stagnated over the past 30 years. The main reasons for OS treatment failure are tumor recurrence and pulmonary metastasis. Chemotherapy is a systemic treatment involving the use of drugs to stop cancer cell growth; its effectiveness largely depends on the efficiency with which chemotherapy drugs are transported to target organs and within specific cells (3). Currently, adriamycin (ADR), used in neoadjuvant chemotherapy (ADR in combination with methotrexate, cisplatin, and ifosfamide), is the standard treatment for patients with OS (4). However, most chemotherapy drugs, including ADR, have toxic side effects. While ADR is the most widely used and effective chemotherapy drug for the treatment of OS, its clinical use is limited by its restricted therapeutic parameters, dose-related cardiotoxicity, and drug resistance. There is an urgent need to develop new treatments to improve OS treatment efficacy and avoid the side effects of chemotherapy. Currently, nanoparticle (NP) drug delivery systems have generated considerable interest as alternative treatment approaches owing to their advantages in enhanced drug penetration and *in vivo* effective circulation time; these reduce the distribution of chemotherapeutic drugs in non-tumor tissues, thus decreasing systemic side effects.

Nanoparticles (NPs) are solid colloidal particles composed of natural synthetic or semi-synthetic polymers, and their diameter (or all three dimensions) ranges from 1 to 100 nm. They usually serve as repositories for nanoparticle systems and carriers for drug delivery systems (5). In addition, some macromolecular nanomaterials are composed of NPs, such as nanotubes, nanosheets, and mesoporous materials, which are used as nanoplatforms for carrying drugs to treat OS (6–8).

Compared with traditional surgery combined with radiotherapy and chemotherapy to treat OS, nanoplatform drug delivery systems are characterized by high drug loading, controllable drug release, remarkable biocompatibility, targeted tumor tissue changes, improved patient compliance, known pharmacokinetics of targeted drugs, and increased blood circulation half-life of drugs. NPs can also carry insoluble drug molecules to enhance their tissue permeability and passively target tumor sites. Furthermore, the modification of superficial active target molecules of NPs can realize targeted delivery of drugs and improve bioavailability at tumor sites (9).

Current research and the development status of OS treatments are summarized in **Table 1**. Compared with normal tissues, tumor tissues are rich in blood vessels, have wide vascular wall gaps and poor structural integrity, and lack a lymphatic drainage system; thus, macromolecular substances and NPs exhibit greater selectivity and higher permeability and retention in tumor tissues than other particle types. The size, quality, surface hydrophobicity, electrostatic effect, magnetic effect, and other physical characteristics of nanomaterials are used to extend the life of chemotherapeutic drug carriers in the blood and their accumulation in pathological parts of the vascular system to achieve passive, targeted drug delivery. In addition, it is possible to modify the surface of NPs through the coupling of specific targeting molecules (e.g., ligands and monoclonal antibodies) Thus, active targeted therapy of pathological cells can be achieved by attaching specific ligands to the surface of chemotherapy drug carriers, thereby improving recognition and binding. For example, Niu et al. reported a functionalized graphene-dendrimeric system loaded with doxorubicin (DOX) *via* Fe_3_O_4_ NPs as a magnetic nanocarrier with high drug-loading capability. They showed that the nanocarrier reduced the toxic side effects of DOX, such as cardiomyopathy, hair loss, leukopenia, thrombocytopenia, and delayed cardiotoxicity, in normal tissues and organs. These results were likely obtained because of improved DOX delivery into OS tissues and enhanced permeability and retention with the use of an external magnetic field (28). In addition, Qiu et al. modified and synthesized tumor-targeting peptide-decorated polypeptide NPs that solved the lack of target specificity of DOX. The tumor-targeting, peptide-decorated, DOX-loaded mPEG-P(Phe-co-Cys) NPs were constructed *via* the ring-opening polymerization of amino acids (29).

**Table 1:** Examples of preclinical studies about different nanoplatform in the treatment of osteosarcoma.

Nano Carrier	Cargo	Target	Mechanism	Strengths	References
**1. liposomes**					
Chol-SS-mPEG/HA-L	DOX	Overexpression of CD44 in OS cells	DOX is targeted for release into OS cells from the Chol-SS-MPEG/HA-L NP triggered by the intracellular GSH response	Combined with chemotherapy drug, nanoparticle administration can enhance anti-tumor activity without causing major side effects	Chi et al. (10)
GEM/CLF co-loaded liposomal formulation	GEM(Gemcitabine) and CLF(Clofazine)	OS cells	This NP uses liposome material to simultaneously load two drugs into specific OS cells *in vivo*	The combined GEM + CLF prolonged the tumor growth inhibition, resulting in the minimal drug dose	Caliskan et al. (11)
Liposome-Encapsulated Curcumin-Loaded	curcumin	OS cells	This 3DP scaffold can not only release curcumin to inhibit osteosarcoma cells, but also promote the proliferation and differentiation of osteoblasts	3DP scaffolds enables CUR to efficiently remove OS cells and promote adhesion, proliferation, and differentiation of healthy osteocytes	Sarkar and Bose (12)
**2. PlOYMER**					
**2.1 Hydrogel**					
SP@MX-TOB/GelMA	Tobramycin(TOB)	OS cells	Under 808 nm near-infrared (NIR) irradiation, thermal ablation can rapidly and effectively kill osteosarcoma cells. Tobramycin (TOB) shows strong antibacterial properties.	Chemotherapy and PTT synergistic treatment	Yin et al. (13)
**2.2 Chitosan**					
ZSM-5/CS/DOX nanodisks	DOX	OS cells	ZSM-5/CS/DOX nanodisks have PH response, high drug release ability in slightly acidic OS environment, and CS and mesoporous ZSM-5 molecular sieves have good biocompatibility and drug delivery efficiency	The nanoplatform can rapidly release DOX under acidic conditions and can avoid DOX leakage under physiological pH	Yang et al. (14)
MCSC scaffolds (a kind of innovative and multifunctional magnetic mesoporous calcium sillicate/chitosan porous scafolds)	DOX	OS cells	The MSCC/DOX scaffold can rapidly release DOX and synergistic hyperthermia under near-infrared light to destroy OS cells. In addition, the scaffold can promote bone marrow stromal cell adhesion, proliferation and osteogenic differentiation to promote new bone regeneration	Chemotherapy and PTT/MTTT synergistic treatment	Yang et al. (15)
**3. inorganic nanomaterials (Nano-composite oxide)**					
**3.1 nano oxide**					
PEG-GOFA/ICG	DOX and TH287 (inhibitor of MTH1)	OS cells	GO can increase the ROS level in tumor cells under near-infrared light irradiation. Mth1 inhibitors can improve the efficacy of Chemo-PDT by inhibiting Mth1 protein and improving the sensitivity of cells to ROS, and promote cell apoptosis and autophagy	Magnetic NPs are safe, non-toxic and can actively target OS cells, and cooperate with PDT to kill OS cells	Huang et al. (16)
**3.2 nano-composite oxides**					
β-TCP–Fe–GO	Fe3O4 magnetic particles	OS cells	b-tcp – Fe – GO magnetic scaffolds inhibited the growth of OS cells by reducing RNA and DNA synthesis and inhibiting protein synthesis. Go can promote the growth, differentiation and osteogenic gene expression of human bone marrow stromal cells	This novel bifunctional biomaterial can not only kill residual bone tumor cells, but also repair bone defects	Zhang et al. (17)
**3.3 Nano metals and alloys**					
Au@AgNRs@BSA	Au and Ag nanoparticals	U-2 OS and Saos-2	This SERS nano-labeled antibody can specifically bind to the overexpressed cells of the recombinant protein receptor. In the SERS mapping experiment of U-2 OS and SAOS-2, it showed high strength, allowing the tumor targeting ligand to easily bind to BSA functional groups. By using the Mica antibody as the targeting ligand, the OS cells can be targeted to activate.	This NP applied to the ultra-sensitive detection of osteosarcoma cells in SERS	Yue et al. (18)
**3.4 Other inorganic nanomaterials**					
**3.4.1 quantum dots**					
2DG-g-GQDs	2-deoxy-D-glucose (2DG)	OS cells	2DG-G-GQDS can synergistic with ionizing radiation to promote the excessive production of ROS, increase mitochondrial damage and significantly increase the oxidative stress response and DNA damage of OS cell lines, so as to realize the sensitization of primary and metastatic OS targeted radiotherapy	The NP has strong tumor targeting performance and penetration efficiency and can cooperate with ionizing radiation therapy	Tung et al. (19)
**3.4.2 Carbon nanotubes**					
SWCNT (Single-walled carbon nanotube)	Oscs induced by TGFβ1	OS cells	SWCNTs decreased the activity of OSCs, significantly inhibited TGFβ1 signal transduction in OS cells, restricted the formation of OS, and prevented the expression of OS markers, differentiation potential, self-renewal ability and chemotherapeutic resistance from reversing to stem cell phenotype.	This NP prevents OS formation at the OSCs level	Miao et al. (20)
**4. Micelles**					
NP-PTX-DOX nanoparticles	PTX and DOX	K7 OS cells	NP-PTX-DOX nanoparticles, as dual-drug, reductive/pH-responsive nanomaterials, can be effectively internalized by K7 OS cells and release PTX and DOX efficiently in response to reduction and acid stimulation.	NP-PTX-DOX NPS has synergistic therapeutic effect, can induce apoptosis of osteosarcoma K7 cells, and has better biological distribution and higher tumor inhibition effect.	Li et al. (21)
CUR loaded ALN-HA-C18 micelles	CUR	OS cells	CUR loaded AlN-HA-C18 micelles showed higher cytotoxic activity against MG-63 cells, significantly improved *in vivo* antitumor activity and reduced systemic toxicity.	The NP can be target CUR to OS cells at a predetermined rate and within a predetermined time, which reduces the drug poisonousness	Xi et al. (22)
**5. Dendritic macromolecules**					
DOX loaded AG/G5 nanogels	DOX	CAL-72 cell	Ag nanogel slowly released DOX, which was effectively absorbed and delivered by CAL-72 cells to exerts anticarcinogenic toxicity. G5 dendrimers can be tracked by fluorescence microscopy once they enter OS cells.	G5 dendritic macromolecule can increase DOX drug loading by three times. Ag gel ensures slow release of DOX in CAL-72 cells, thus enhancing the anticancer effect of the drug and reducing the systemic toxicity of the drug.	Goncalves et al. (23)
**6. Nanocapsule**					
IFS-LNC (the Ifosfamide-loaded-lipid-core nanocapsules)	IFS (Ifosfamide)	OS cells	The endocytic uptake of IFS-LNC nanocapsule by MG63 cancer cells accelerated the destruction of acidic endosomal vesicles of the nanocapsule and accelerated the release of IFS into the cytoplasm. The apoptosis of OS cells was increased by increasing the expression levels of Caspase-3 and Caspase-9 in MG63 cells	IFS-LNC has a narrow particle size distribution, so it has a high drug loading capacity. IFS inhibits DNA replication through cross-linking DNA chains and destroys OS cells at the genetic level	Wang et al. (24)
AON-loaded NPs(AON : Antisense oligonucleotides)	AON	Ewsflil-1 oncogene	AON-loaded NPS cationic surfactant CTAB can penetrate the endosomal membrane, thereby inducing more effective cytoplasmic delivery of AON and promoting mRNA specific down-regulation of EWS-FLI-1 in a rat model of OS	The application of AON loaded nanocapsules overcomes the disadvantages of easy degradation, short biological life and limited cell uptake of AON, which makes AON more effective in intracellular delivery and anticancer effect.	Maksimenko et al. (25)
**7. Exosomes**					
EXO-DOX	DOX	OS cells	The acidic environment such as the late endosome and lysosome of OS cells can accelerate the release of Exo-Dox, making the cell absorption efficiency of Exo-Dox higher than that of free DOX	Compared with free DOX, Exo-Dox has better cell uptake and antitumor effect on OS, and has a selective antitumor effect on cardiomyocytes.	Wei et al. (26)
Hic-5-EXO	EXO	Hic-5 gene	Hic-5-Exo effectively inhibited the expression of Hic-5 gene in MG-63 cells, thus inactivating Wnt/β-catenin signal, effectively inhibiting the proliferation of OS cells at the gene level.	Proliferation and apoptosis of OS cells were effectively inhibited at the gene level	Sha et al. (27)

Compared with other tumors, the complex cytology of OS is not sensitive to conventional radiotherapy and chemotherapy, and the side effects of multiple chemotherapeutic drugs are very serious (6). In addition, a large number of therapeutic studies on OS nanoplatforms have shown that, compared with other tumors, nanoplatform drug-loading has an urgent need for clinical transformation and bright prospects in the treatment of OS (11, 13). The use of NPs enhances the bioavailability and selectivity of chemotherapy drugs, significantly improves the therapeutic effect of traditional treatment methods, shortens treatment time, improves patient compliance, reduces medical costs, and results in innovative changes in OS drug treatment. In this review, we introduce recent scientific advancements made in different types of nanoplatform drug delivery systems, which are usually divided into the following categories: liposomes, polymeric NPs, inorganic NPs, nanomicelles, dendrimers, nanocapsules, and exosomes. Each of these drug carriers are discussed in detail in the subsequent sections.

## 2 Research Advances in Nanoplatforms for the Treatment of OS

### 2.1 Liposomes

Liposome NPs were the first nanodrug delivery system to be transitioned from concept to clinical application. They are highly biocompatible, biodegradable, non-toxic, spherical vesicles consisting of a hydrophilic core and a lipid bilayer, and they can be mass-produced in the 50–150 nm size range (30). Liposomes are one of the most promising NP carriers because of their ability to protect loaded drugs from biodegradation, prolong the plasma half-life of drugs, improve drug stability and bioavailability, and sustain *in vivo* release and targeting (31).

**Figure 1:**
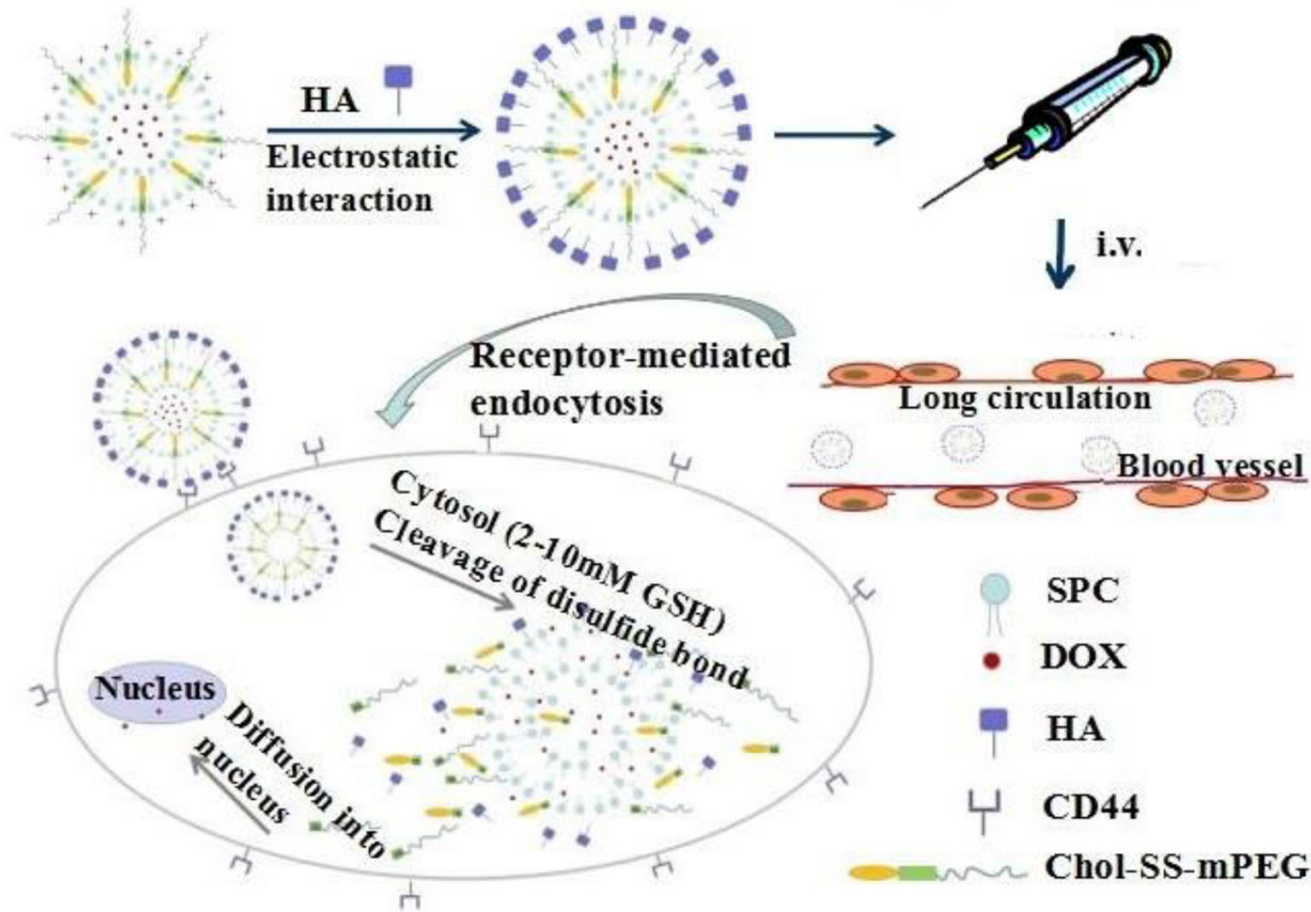
Chol-SS-mPEG/HA-L designed for long circulation *in vivo* followed by receptor-mediated endocytosis and GSH-triggered cytoplasmic DOX release. Reproduced from (10) with permission from J Control Release.

Chi et al. designed and synthesized a novel, ideal polyethylene glycol (PEG) bound to cholesterol *via* disulfide bonds, where hyaluronic acid (HA) was further coated with a hydrophilic shell and the CD44 ligand on the surface of the liposome. They successfully synthesized a redox-sensitive and CD44 targeting NP named Chol-SS-MPEG/HA-L (10). Cho-SS-MPEG/HA-L NPs are internalized into CD44-overexpressing OS cells through CD44-mediated endocytosis. Upon entering the cell, DOX is released from the Chol-SS-MPEG/HA-L NPs due to PEG detachment triggered by the intracellular glutathione (GSH) response. Chol-ss-mpeg/HA-L NPs loaded with DOX had a longer circulation time *in vivo*, and the NPs could target OS cells through CD44, which significantly improved the inhibitory effect on OS cells, reduced the toxicity of DOX to non-tumor tissues, and maximized the anti-OS effect of DOX (**Figure 1**).

In recent years, new strategies for combining two or more antineoplastic drugs to reduce side effects, while ensuring effective dosage administration in cancer treatment, have received increasing attention. Gemcitabine (GEM), which inhibits DNA synthesis, is one of the most commonly used first-line cancer drugs in many cancer treatments (32). Nevertheless, it is limited by having a short half-life, being too hydrophilic to cross cell membranes, and causes hepatotoxicity and nephrotoxicity. Clofazine (CLF) inhibits Wnt signaling pathways, which are overexpressed in many cancers, including OS (33). Caliskan et al. utilized the selective localization of GEM and CLF on liposome structures to prepare a new GEM/CLF co-loaded liposome preparation with a synergistic effect of the two drugs (11). Compared with single-agent chemotherapy, GEM/CLF co-loaded liposomes exhibited a higher percentage of tumor cytotoxicity, apoptotic cell ratio, caspase-3 activity, and mitochondrial membrane depolarization. This NP takes advantage of the liposome NP to simultaneously load two drugs to target specific OS cells *in vivo*, without increasing the dose of vectors and chemotherapeutic drugs, greatly improving the OS therapeutic effect. Furthermore, Chen et al. synthesized sali-entrapped lipid-polymer NPs labeled with CD133 and EGFR aptamers (CESP) to target OS and osteosarcoma cancer stem cells (CSCs) (34). The binding of EGFR aptamers on CESP not only increases the targeting of CESP CD133-OS cells, but also further increases the targeting of CESP to CD133+. The results of *in vitro* experiments showed that CESP significantly inhibited OS activity compared with free salinomycin, non-targeted, or single-targeting NPs. In OS tumor-bearing mice, CESP showed the best inhibitory effect on tumor growth compared with controls.

**Figure 2:**
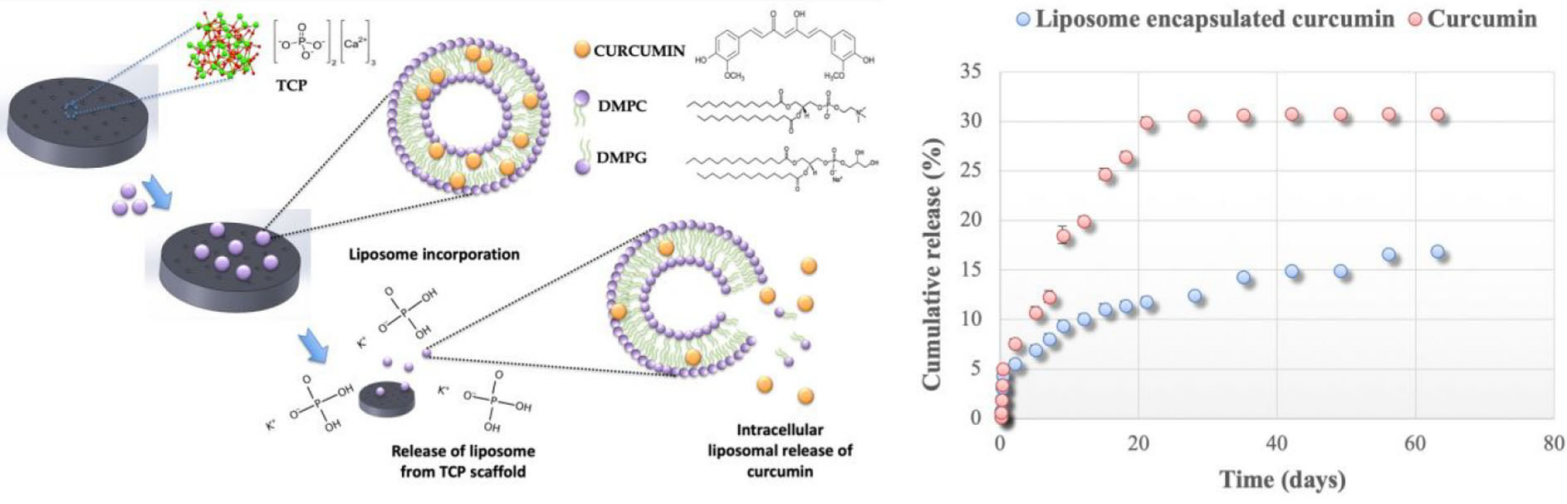
3DP scaffolds containing DMPC and DMPG formed liposomes loated with curcumin. In the presence of a buffer medium or an extracellular medium, liposome released, followed by curcumin released from the liposome. Reproduced from (12) with permission from ACS Appl Mater Interfaces.

Bone is the second most common requirement in the world of transplanted organs; each year, more than 2 million cases of surgery using bone grafts or bone substitute materials are carried out to treat bone defects (35). After bone tumor resection, failure to completely remove bone tumor cells frequently leads to their continued proliferation, thus leading to bone tumor recurrence. Furthermore, huge bone loss, especially in patients with immature bone defects, may cause limb length inconsistencies and abnormal gait and significant limb dysfunction, resulting in great physical and mental pain to the patient. Traditional bone prosthesis has disadvantages, such as rejection, inflammation, infection, and patient intolerance. Therefore, bioactive three-dimensional (3D) scaffolds used for the synthesis of bone graft substitutes have been getting increasing attention in bone tissue engineering research owing to their good biocompatibility. Biological bone 3D scaffolds are composed of biodegradable materials; their mechanical properties and the implanted tissue are designed by 3D printing technology through computer aided design (36). This allows for the precise control of microscopic and macroscopic structures, interconnected pore geometry, targeted drug delivery, and repair of bone through tissue engineering. In another words, the 3D technology not only controls the size of the pores, but also allows for the preparation of individual shapes to meet various bone surgery requirements, and also the delivery of chemotherapeutic drugs to prevent recurrence of residual bone tumor cells (37, 38). A high precision is needed for surface pores or vascular channels in the geometric 3-D framework; this can significantly affect the adhesion proliferation of bone tissue cells or maturation, which is of great significance for bone remodeling and repair. An example of this strategy was reported by Sarkar et al., who successfully encapsulated curcumin in liposomes and combined it with 3D-printed (3DP) calcium phosphate (CaP) scaffolds with designed porosity (**Figure 2**) (12). The scaffold, with its designed shape and interconnected porosity, provides new tissue growth sites and mechanical support for cell growth and attachment through mechanical interlocking between the surrounding host tissue and the scaffold. Curcumin released from the 3DP scaffold showed significant tumor cytotoxicity to OS cells and extended *in vivo* circulation time, reduced systemic toxicity, and promoted osteoblast cell viability and proliferation. This dual-function curcumin liposome scaffold can efficiently remove OS cells and promote the adhesion, proliferation, and differentiation of healthy bone cells in the porous scaffold, providing a promising therapeutic strategy for the treatment of bone defects after tumor resection.

Despres et al. found that the surface charge of liposome NPs can affect the immune response (39). Villegas et al. also reported that liposome NPs are easily phagocytosed by mononuclear macrophages and have low stability in serum protein and *in vivo* (40). In addition, the instability of traditional liposomes, insufficient circulation time *in vivo*, drug leakage, large particle size, immune stimulation, and complement activation limit the further clinical application of liposomes (41). However, PEG has been used to modify liposome nanomaterials to reduce particle aggregation through steric hindrance stabilization, thus enhancing liposome *in vivo* stability, prolonging *in vivo* circulation time, and increasing targeted accumulation in disease sites (42).

### 2.2 Polymeric NPs

Polymeric NPs are a new type of nanomaterial that have attracted increasing attention in cancer diagnosis and treatment because they can be modified by chemotherapeutic drugs, antibodies, or nucleic acid ligands, and they can recognize cancer markers in cancer cell membranes or inside cells (43). They can be used to target the transport of drugs or nucleic acids during chemotherapy or gene therapy. Enzymatic reactions, ultrasound, magnetism, electricity, light, pH response, and REDOX response can all act as driving forces for drug release from polymeric NPs *in vivo*, allowing them to be targeted for transport to tumor cells (44). Polymeric NPs can be simply divided into hydrogels and chitosan, among other types.

#### 2.2.1 Hydrogels

Hydrogels are crosslinked hydrophilic polymeric NPs synthesized in natural or synthetic forms (45). When hydrogels are activated or modified by magnetic materials or fluorescent materials, drugs can be encapsulated and released continuously and sustainably to the targeted tissue. In addition, hydrogels are excellent filling materials for bone tissue regeneration because of their remarkable shaping ability, stable structure, and mechanical support ability (46). Hydrogels have broad application prospects in the biomedical field owing to their excellent biocompatibility, degradability, good drug loading ability, REDOX, and pH responsiveness.

There is still a high risk of tumor recurrence, postoperative infection, and massive bone loss after the resection of OS. Traditional treatment methods rely on postoperatively implanted orthopedic materials to fill bone defects, but they are unable to inhibit the growth of residual OS cells and prevent bacterial infection (47). Photodynamic therapy (PDT) is a new, minimally invasive treatment that activates only specific tissue areas exposed to light and, when combined with nanotechnology, it is more effective than traditional treatment methods (48).

**Figure 3:**
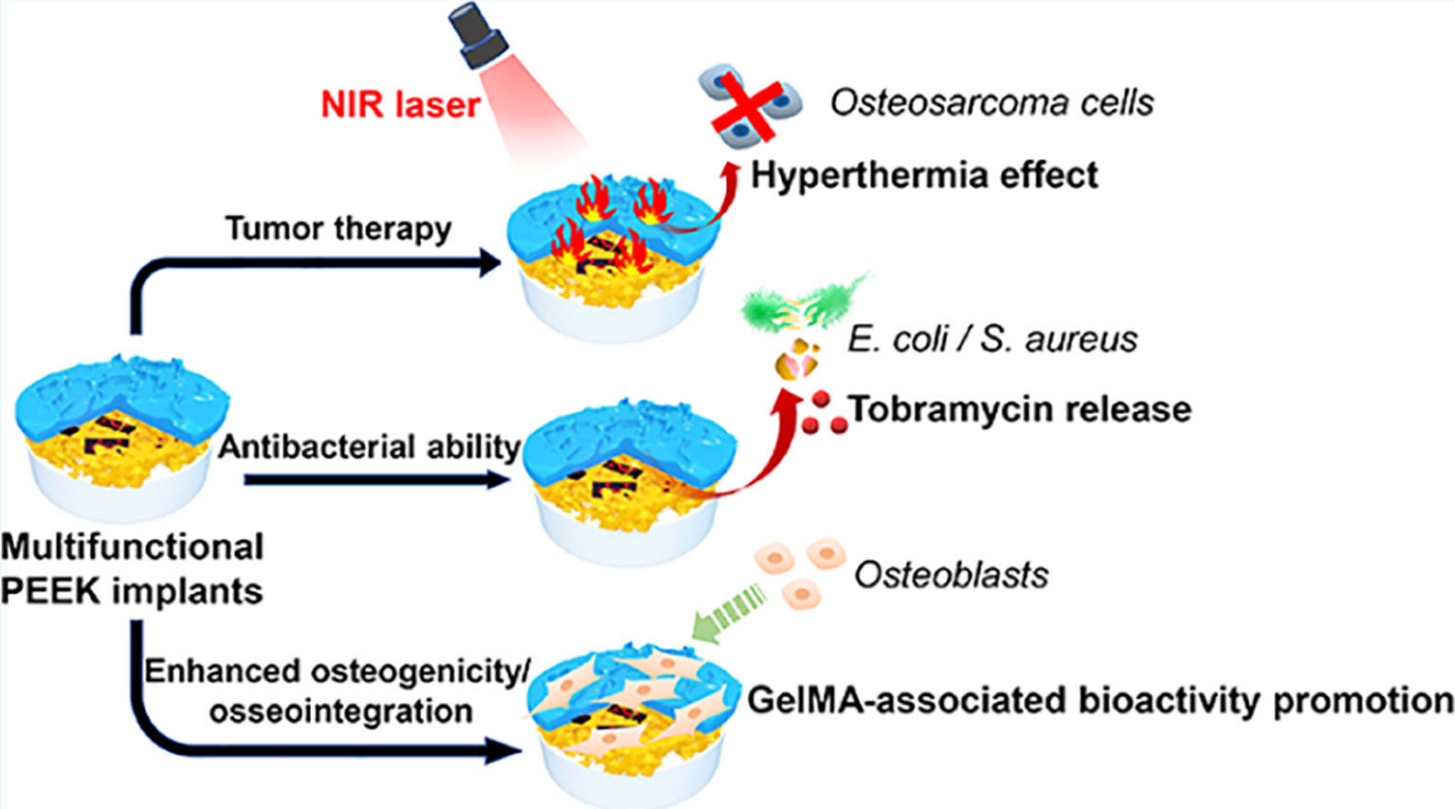
Under 808 nm near-infrared (NIR) irradiation, SP@MX-TOB/GelMA multifunctional implants can effectively kill OS cells in conjunction with photothermal effect. In addition, SP@MX-TOB/GelMA loaded TOB showed good antibacterial properties. More importantly, the multifunctional implant has excellent cellular compatibility and osteogenic ability. Reproduced from (13) with permission from Curr Med Chem.

Yin et al. developed a novel multifunctional nanoimplant, SP@MX-TOB/GelMA, with excellent biocompatibility, wide light absorption capacity from UV to near-infrared (NIR) light, and high photothermal conversion efficiency (13). Under 808 nm NIR irradiation, the thermal ablation effect can effectively eliminate OS cells and promote bone tissue regeneration through heat therapy (**Figure 3**). In addition, SP@MX-TOB/GelMA has a large surface area and a large number of negatively charged particles that can carry highly effective antibacterial agents (such as tobramycin) to prevent bacterial invasion and infection, showing strong antibacterial properties against both gram-negative and gram-positive bacteria. More importantly, multifunctional implants have superior cytocompatibility and osteogenic ability in terms of cell replication, diffusion, calcium matrix mineralization, and *in vivo* bone integration. Consequently, this photothermal-controlled multifunctional implant can not only eliminate OS cells and fight against pathogenic bacteria, but can also enhance osteogenic ability, providing a promising measure for the treatment of tissue defects after OS resection.

Despite the many benefits of hydrogel NPs, certain disadvantages limit their use in clinical applications. For example, pure hydrogels often have suboptimal strength, compressibility, and elasticity (49), and the inherent hydrophilicity of hydrogels may complicate the high loading and sustained release of certain drugs. In addition, the diameter and number of NPs play a crucial role in the formation of hydrogels, and research shows that NPs with a diameter exceeding 100 nm cannot form a stable hydrogel (50).

#### 2.2.2 Chitosan

Chitosan, which is composed of β- (1–4)-linked glucosamine units, can be used to construct nanospheres and NPs for drug delivery systems (51). The sizes range between 1 μm and 500 μm. Chitosan is strong, semi-permeable, well-organized microstructurally, and exhibits a high affinity for biofunctionalized bioactive agents and drugs. Owing to its remarkable biocompatibility, slow-releasing potential, biodegradable properties, good mechanical stability, low toxicity, low production cost, and closeness to the physiological conditions of film-forming ability, it has gradually become an effective drug delivery system (DDS) for chemotherapy drugs and gene therapy (52). Chitosan can be applied to advanced 3D printing to generate bone scaffolds with highly complex geometric shapes, accelerating bone tissue regeneration.

Fan et al. first prepared mesoporous zeolite/chitosan core-shell nanodisks loaded with DOX (ZSM-5/CS/DOX nanodisks) for the treatment of OS (14). ZSM-5/CS/DOX nanodisks have the following advantages (1): mesoporous and microporous ZSM-5 nanodisks are conducive to improved drug loading rate (2); the core-shell layer of the mesoporous ZSM-5 nanodisk enables the pH response of the nanocomposite, and the drug release rate is higher in a slightly acidic tumor environment than in normal tissue (3); both CS and mesoporous ZSM-5 zeolites have good biocompatibility; and (4) reduced degradation of DOX and slower release of DOX delivery at the tumor site are observed. This method showed that a slow release of DOX in MG-63 cells enhanced apoptosis of the cells, and ZSM-5/CS/DOX NPs showed no systemic toxicity. The pH-responsive ZSM-5/CS/DOX nanodisks released a higher dose of DOX in a slightly acidic tumor environment. Compared with free DOX, this controlled release ensured an effective blood drug concentration and a greater ability to inhibit MG63 cells, while showing a relatively strong anti-OS effect. At the same time, it reduced the elevation of CK and MDA, reducing the cardiac toxicity of DOX. Noteworthy, ZSM-5/CS/DOX NP-treated mice did not show any visible adverse effects. This study proved that ZSM-5/CS/DOX nanodisks are promising pH-responsive drug carriers for the targeted treatment of OS.

**Figure 4:**
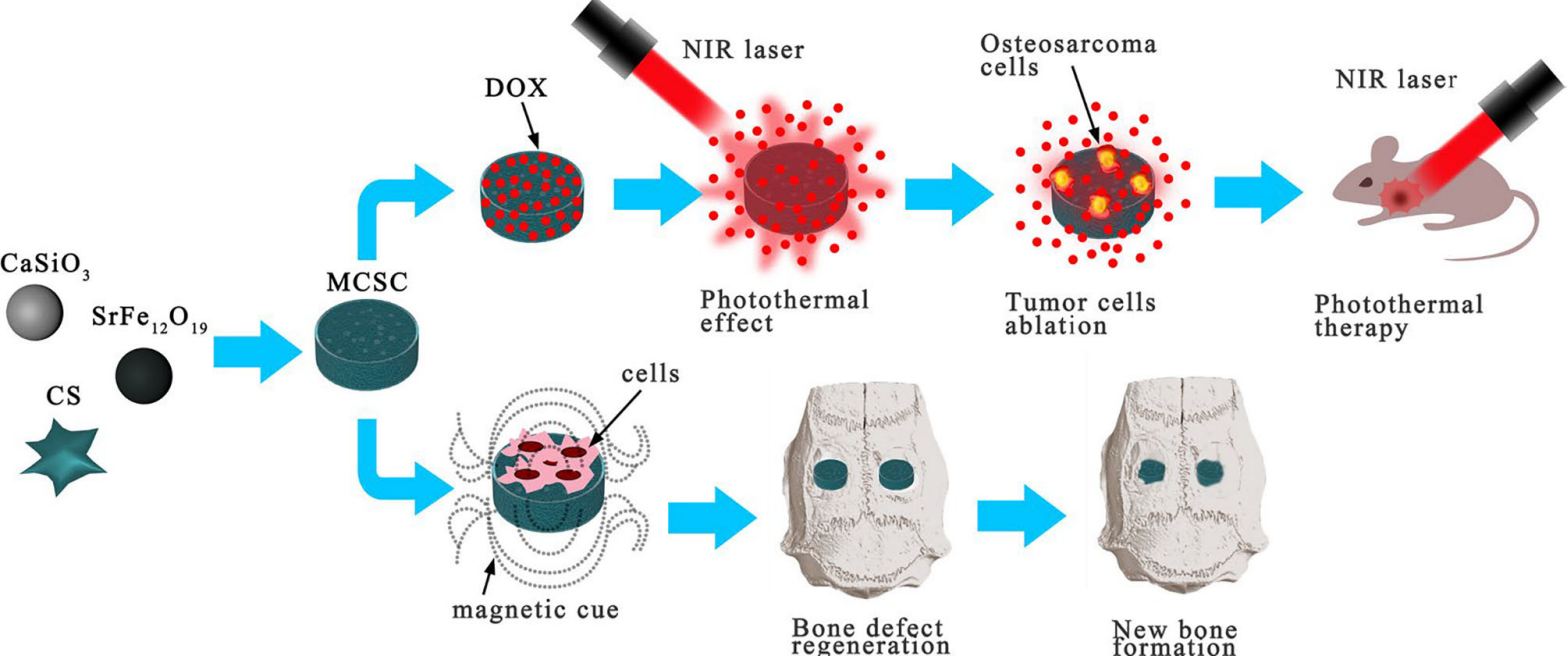
MCSC scaffolds were composed of M-type ferrite particles and mesoporous calcium silicate microspheres dispersed in chitosan membrane. The scaffolds showed good drug delivery performance under NIR laser irradiation, and the MCSC scaffolds also had excellent ability to promote new bone regeneration. Reproduced from (15) with permission from Sci Rep.

The development of multifunctional biomaterials to repair bone defects and inhibit tumor recurrence after the resection of OS continues to be a huge clinical challenge. In recent years, calcium silicate and chitosan have been widely used as bone-forming materials or scaffolds because of their remarkable biocompatibility and bioactivity (53). SrFe12O19 is an m-type ferrite material with strong intrinsic magnetic properties (54). Yang et al. introduced a novel multifunctional magnetic mesoporous calcium silicate/chitosan porous scaffold composed of SrFe12O19, CaSiO3, and CS that can carry the chemotherapy drug DOX (15). The mesopores in the CaSiO3 microspheres are conducive to drug delivery and exhibit remarkable NIR absorbance, photothermal conversion efficiency, and thermal conductivity. SrFe12O19 particles can improve the conversion effect of photothermal therapy. Under NIR laser irradiation, the local temperature of the photothermal particles increased to 42–50°C, and DOX was rapidly released from the MCSC/DOX scaffold. The MCSC/DOX scaffold rapidly eliminated OS cells through the synergistic effect of DOX drug release and thermotherapy ablation. Evidence has indicated that an injectable chitosan formulation aids in healing bone defects by means of structural modification. Culturing human bone marrow stromal cells (hBMSCs) with chitosan, which is water-soluble, promoted the expression of BMP-2, p-Smad1/5, and Runx2 at the protein level, and improved the adhesion, proliferation, and osteogenic differentiation of new bone through the BMP/Smad signaling pathway. Thus, it is implied that MCSC/DOX scaffolds are capable of aiding the healing of bone defects (15) (**Figure 4**). Overall, the clinical application of MCSC/DOX scaffolds as a promising NP for the treatment of OS warrants further research.

However, the clinical application of chitosan as a cationic nano-polymer continues to exhibit certain challenges, such as chitosan being insoluble in water, most organic solvents, and physiological pH conditions; thus, it is not ideal for certain drug loading systems (55). Moreover, chitosan nanomaterials may have a mild toxic effect on the liver (56). Scientists have found that they can improve the physical and chemical properties of chitosan by introducing chemical modifications.

### 2.3 Inorganic NPs

Inorganic NPs mainly include nano-oxides, nano-composite oxides, nanometals, and alloys (57). Inorganic NPs provide a multifunctional platform for biomedical applications, such as drug delivery, biosensing, bioimaging, disease diagnosis, and bone defect repair, owing to their excellent mechanical properties (58).

#### 2.3.1 Nano-Oxides

Nano-oxides refer to nano-sized oxides, such as nano titanium dioxide, nano silicon dioxide, nano zinc oxide, nano alumina, nano zirconia, nano cerium oxide, and nano iron oxide (59). The basic technical parameters of nano-oxides include particle size, content, specific surface area, pH, and content of some metal components. Nano-oxides have abundant adsorption sites and surface functional groups that can maximize their drug-loading capacity (60).

**Figure 5:**
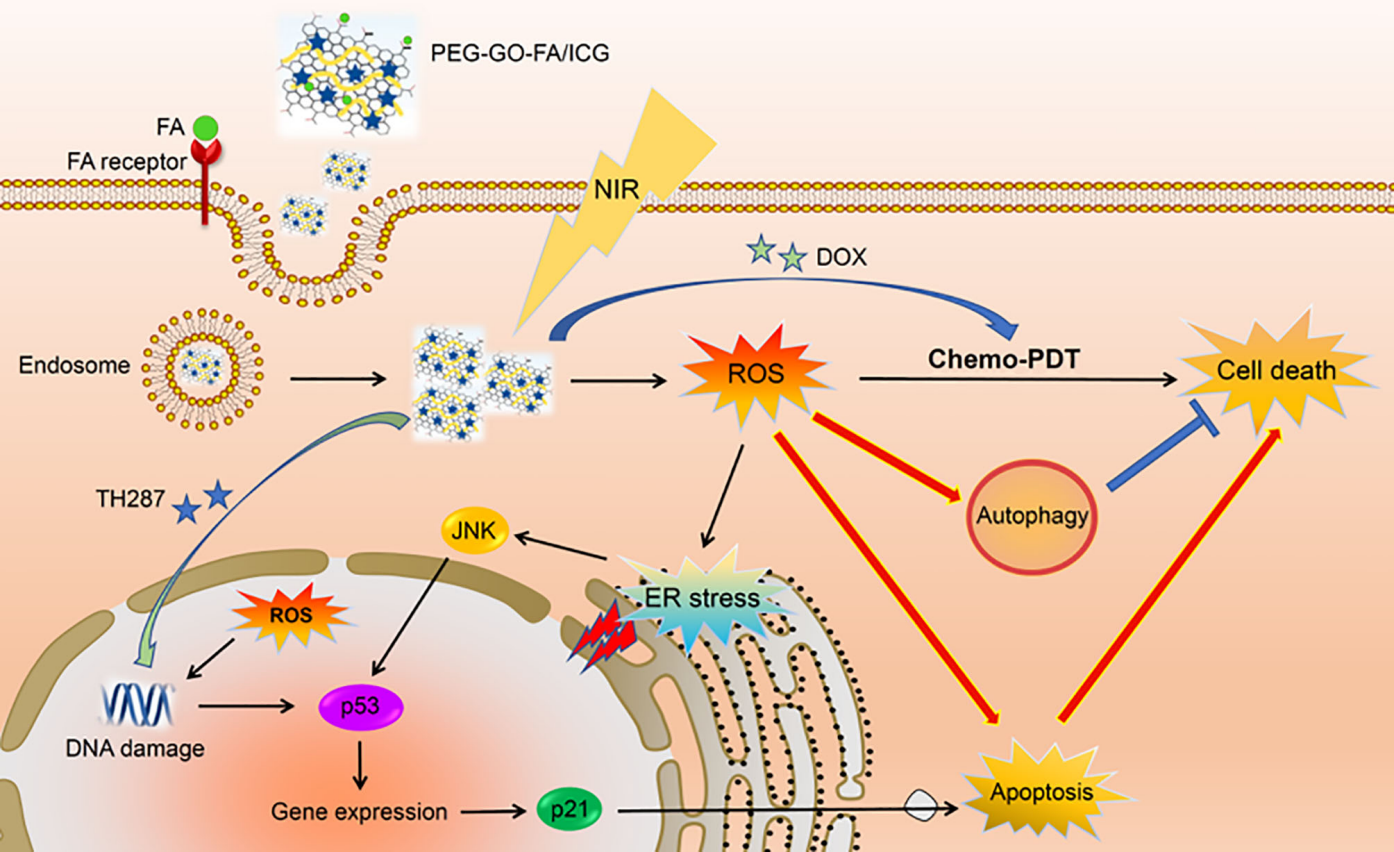
PEG-GOFA/ICG nanoplatform is absorbed by OS cells through FA receptor-mediated endocytosis. Under NIR irradiation, PEG-GOFA/ICG nanoplatform has CHEMO-PDT synergistic effect to promote intracellular ROS production and release MTH1 inhibitor TH287 to enhance apoptosis induction of OS cells. Reproduced from (16) with permission from Acta Biomater.

PDT relies on highly toxic reactive oxygen species (ROS) produced by photosensitizers in the treatment of non-invasive cancer. The wavelength of the NIR laser ranges from 700 to 1000 nm, and NIR has strong tissue penetration (61). Graphene oxide (GO) has a strong infrared light absorption capacity and exhibits photothermal effects when irradiated at 808 nm (62). Hence, GO NPs loaded with drugs and photosensitizers can locally exert a combined chemical-photothermal-photodynamic effect to enhance the antitumor effect of traditional drugs. The surface of OS cells overexpresses folic acid (FA) receptors and presents with a slightly acidic environment with a pH of 6.5–7.2. MutT homolog 1 (MTH1) protein is a DNA oxidative damage repair protease; therefore, the inhibition of MTH1 protein function may be a novel anti-tumor strategy. Huang et al. constructed PEG-GOFA/ICG NPs loaded with the chemotherapy drugs DOX and TH287(MTH1 inhibitors) (16). Using ICG as a photosensitizer, PEG-GOFA/ICG NPs exhibited superior chemo-photodynamic (CHEMO-PDT) therapy capability to GO alone and the ability to respond to the slightly acidic environment of the tumor. PEG-GOFA/ICG NPs control the targeted release of DOX and TH287 in the slightly acidic environment of OS, thereby enriching the drug locally in the OS tissue and reducing its toxic effect on normal cells. Moreover, GO can increase ROS levels in tumor cells under NIR light. MTH1 inhibitors enhance CHEMO-PDT efficacy by inhibiting the MTH1 protein, increasing cell sensitivity to ROS, and promoting apoptosis and autophagy of OS cells, thus greatly enhancing the anti-OS effect (**Figure 5**). Recent advances in the development of magnetic NPs have demonstrated their non-toxicity and good photothermal conversion efficiency. Lu et al. produced magnetic NP-modified porous scaffolds, which not only significantly promoted stem cell osteogenic differentiation by activating the BMP-2/Smad/Runx2 pathway, but also increased the local temperature of OS tissue by 42~50 °C under NIR light, which strengthened the thermotherapeutic effect on the tumor (63). In another study, novel magnetically targetable SPIONs, based on magnetite (Fe_3_O_4_) NP surface modified with β-cyclodextrin (CD), were loaded with paclitaxel (PTX). The Fe_3_O_4_@β-CD/PT NPs exhibited a high PTX loading ability and strong magnetic response under an applied magnetic field, and a significantly increased affinity for tumoral cells (64). Considering these pieces of evidence, magnetic NPs could be superior to traditional chemotherapy because of their advantages in targeted drug delivery, delivering a local hyperthermia, and magneto mechanical effect on cancerous cells.

Mesoporous silica nanoparticles (MSNs) are widely used as drug carriers due to their excellent physical and chemical properties, such as good biocompatibility, targeting subcellular compartments, ease of different functionalizations, large drug loading capacity, easy surface modification, adjustable size, and good thermal/chemical stability (65). In 2018, Liu’s group directly constructed targeted mesoporous silica-coated bismuth sulfide NPs (Bi2S3@MSNs) equipped with DOX for combined photothermal therapy–chemotherapy for the diagnosis and treatment of highly malignant OS (66). The results indicated that whether these NPs were used for real-time CT imaging or for combination to diagnose and treat highly malignant OS, they exhibited extremely high drug encapsulation efficiency (99.85%) and excellent PTT efficiency (36.62%). It is worth mentioning that the *in vivo* experimental results showed that the RGD–Bi2S3 @MSN/DOX NPs synthesized by Liu et al. combined with near-infrared radiation can effectively ablate malignant OS and prevent its recurrence (66). Similarly, Hu et al. developed a new type of mesoporous silica-coated gold nanostar (GNS@MSNs-FA) modified by folic acid (FA) with the Chinese medicine lyricine (Ly) NPs (67). In summary, the researchers utilized gold nanostars (GNS) with multiple sharp branches as the core of the nanocomposite, employing MSNs to coat the core-shell GNS@MSNs with Ly, and then modified the GNS@MSNs with amino groups, which played a role in targeting tumor cells. GNS@MSNs-FA/Ly combined with NIR irradiation can promote high-level ROS production by inducing mitochondrial dysfunction and effective endoplasmic reticulum stress. Both *in vitro* and *in vivo* studies have shown that the combination of GNS@MSNs-FA/Ly and NIR irradiation of NPs has a better OS inhibitory effect. In addition, the nanocomposite showed excellent anti-tumor properties in OS tumor-bearing mice without significant toxicity.

#### 2.3.2 Nano-Composite Oxides

Owing to their excellent comprehensive properties, especially their designability, nano-composite oxides have been widely used in medical, aerospace, and national defense fields, among others (68). Furthermore, the recent development of nanocomposite oxides has been very rapid. The primary research directions include nano-graphene oxide materials, carbon tube nanocomposites, and tungsten copper nanocomposites.

Magnetothermal treatment is a type of hyperthermia therapy that can affect the fluidity and stability of cell membranes and inhibit protein synthesis by reducing the intracellular synthesis and aggregation of RNA and DNA molecules, thereby significantly impacting tumor cells (69). GO has remarkable biocompatibility and thermal conductivity and can promote the growth, differentiation, and osteogenic gene expression of human bone marrow stromal cells (70). Zhang et al. prepared magnetic scaffold B-TCP-Fe-GO NPs by modifying the surface of a porous scaffold with a small amount of magnetic particles (17). The magnetic properties of the scaffold can be adjusted by controlling the number of Fe_3_O_4_ particles on the surface of the scaffold, which directly leads to superior magnetic and high-temperature performance of the magnetic scaffold. When B-TCP-FE-GO scaffolds are used in the treatment of OS the following have been observed (1): in bone tissue engineering, magnetic scaffolds can meet the needs of normal bone tissue cell proliferation and differentiation (2); the temperature of the magnetic scaffold can be adjusted to 50–80 ℃ in a short time, which effectively kills MG-63 OS cells; when the remaining tumor cells are killed, the tumor recurrence rate is greatly reduced (3); under an alternating magnetic field, the temperature range of the scaffolds can reach a wider range by controlling the magnetic field intensity and Fe_3_O_4_ content *via* the osteogenic activity maintained by the modified magnetic scaffolds; and (4) B-TCP-FE-Go scaffold can significantly stimulate the proliferation and bone-related gene expression of rBMSCs. B-TCP-FE-GO scaffolds with good magnetic properties and osteogenic ability have great application prospects in the treatment and regeneration of bone defects caused by OS.

#### 2.3.3 Nanometals and Nanoalloys

New metallic nanomaterials, including Ti, Mg, Zn, and alloys, have excellent strength and stress absorption capacity, which can overcome the shortcomings of the low mechanical properties of polymeric NPs and brittleness of bioceramics. They have gradually drawn attention as material for the diagnosis and treatment of OS (71).

Recently, surface-enhanced Raman spectroscopy (SERS) has attracted extensive attention owing to its highly sensitive detection of electromagnetic field amplification of local surface plasmons. Gold (Au), silver (Ag), and copper (Cu) are classical SERS substrate materials, and they all have local surface plasmon resonance (LSPR) within the range of visible and infrared wavelengths (72). Because of its mild photo-bleaching, minimal peak over-lapping, excellent spectral specificity, and enhanced signal-to-noise ratio, SERS has become an alternative tool for biological imaging. SERS can detect very low concentrations of analytes, even single molecules; however, owing to the lack of signal reproducibility and quantitative function, the wide application of SERS *in vitro* and *in vivo* imaging is limited. The intensity of the Raman signal enhancement is related to the material, shape, size, and aggregation state. Yue et al. developed a new type of ultrasensitive biocompatibility detector, Au@AgNRs@BSA, which uses a stable SERS substrate and can successfully be applied to the SERS hypersensitive detection of OS cells *in vitro* and *in vivo* (18). Au@Ag bimetallic NPs exhibited higher SERS activity than pure Au or Ag NPs. The results showed that the SERS nano-labeled antibody could bind the recombinant protein receptor, showing high intensity in the SERS mapping experiment of U-2 OS and SAOS-2. This SERS substrate platform allows tumor-targeting ligands to easily combine with bovine serum albumin protein (BSA) functional groups. By using MICA antibody as the targeting ligand, OS cells could be targeted and activated in animal models, leading to specific delivery of chemotherapy drugs to cancer cells, which is important for potential therapeutic applications in OS.

However, the properties of nanomaterials that cause DNA damage and mutations also cause them to have unpredictable effects on the body, thereby resulting in genotoxicity and carcinogenicity (73). This has limited the clinical application of nano-metals and their alloys.

#### 2.3.4 Other Inorganic NPs

The lack of effective means for rapid and accurate diagnosis of OS at an early stage to prevent the metastasis of OS, especially pulmonary metastasis, is undoubtedly fatal to patients with OS. Inorganic NPs such as magnetic and gold NPs, ZnO/Pt-Pd (platinum-palladium alloy), graphene, and quantum dots (QDs) have been reported for disease detection and tracking. In addition, they can also be applied to efficiently deliver drugs or therapeutic molecules to specific organs, tumor imaging, sensitive detection of tumor metastasis, and accurate tracking of host cells.

##### 2.3.4.1 Quantum Dots

Owing to OS radiation resistance, which often necessitate high doses of radiation in radiation schemes, and off-target doses, radiation treatment cannot guarantee the complete elimination of metastatic tumors. Furthermore, it may adversely affect and damage unrelated body organs and the immune system. Therefore, the use of local tumor radiotherapy sensitization agents to enhance regional radiation affects is considered one of the most promising strategies (74). In one study, Tung et al. designed a nanoscale radiosensitizer, 2DG-GQDs, by grafting 2-deoxy-D-glucose (2DG) onto graphene quantum dots (GQDs) (19). NP-based radiosensitizers can selectively accumulate in tumors by enhancing osmosis and retention (EPR) effects, thus minimizing adverse effects. Moreover, these radiosensitizers can synergistically interact with ionizing radiation to promote ROS overproduction and the formation of DNA strain breaks, and increase mitochondrial damage and the oxidative stress response in OS cell lines, thereby sensitizing primary and metastatic OS-targeted radiotherapy and reducing OS cell migration. Compared with radiotherapy alone, 2DG-G-GQDS reduced the risk of distant lung metastases by 95%. The successful use of 2DG-G-GQDS demonstrates that QD material can promote successful tumor-targeted radiation to tumors and enhance the efficacy of radiotherapy.

##### 2.3.4.2 Carbon Nanotubes

Cancer stem cells (CSCs), a subpopulation with stem cell-like properties and the generation of heterogeneous lineages of cancer cells in solid tumors, are believed to play a pivotal role in the development of metastatic drug resistance or recurrence of tumors and are closely related to the progression of OS (75). TGFβ1 is a potent induction factor that induces and maintains CSC characteristics; its overexpression creates a favorable tumor microenvironment for the progression and metastasis of OS (76). Traditional cancer therapies focus on removing cancer cells, but not sustaining a lasting therapeutic response because they cannot kill CSCs effectively. Accordingly, strategies specifically targeting CSCs and the TGFβ1 signaling pathway have become one of the most promising and innovative approaches for the treatment of OS. SB431542 (TGFβR1 inhibitor) can significantly block the TGFβ 1-induced CSCs formation pathway (77). Single-walled carbon nanotubes (SWCNTs) are promising for biomedical applications, such as biosensor disease diagnosis and treatment (78). The hollow interior and non-covalent π–π packing structure of CNTS allow for the loading of drugs. The special physical properties of CNTs, such as photoluminescence with strong NIR absorption and photothermal conversion ability, make them suitable for use as contrast agents in tumor imaging, tracking, and photothermal therapy. Miao et al. found that SWCNTs can rapidly target TGF β1-induced OS stem cells (OSCs, CSCs in OS) *in vivo*. SWCNTs were internalized into OS cells even under hypoxic and acidic conditions. They significantly inhibited TGF β1-induced OS dedifferentiation *in vivo* and blocked the ability of OS markers to express differentiation potential self-renewal (colony formation). In addition, SWCNTs can inhibit the angiogenesis of OS by reversing the chemotherapeutic resistance of the stem cell phenotype, thus further inhibiting OS cell proliferation and tumor growth. Miao et al. demonstrated the potential of SWCNTs to reduce the incidence of OS and reduce the OSC population in OS by blocking the TGF β1 signaling pathway, thus confirming the potential of SWCNTs as OSC inhibitors in OS treatment (20).

### 2.4 Nanomicelles

Nanomicelles are nanoscale aggregated colloids formed in solution by the self-assembly of amphiphilic polymers (79). Their hydrophobic cores allow for the encapsulation of poorly water-soluble drugs. Nanomicelles are typically smaller than other NPs, which prevents their recognition by the reticuloendothelial system (RES) and macrophages, thereby facilitating passive targeting of solid tumors and tissue penetration, and allowing longer circulation times for drugs (80). Furthermore, the simple preparation and sterilization process, as well as remarkable solubilization performance, enable nanomicelles to overcome problems related to drug delivery, such as low water solubility, inability to cross the biological barrier, and low permeability.

More recently, Li et al. synthesized a REDOX/PH dual-responsive nanocolor mPEG-PaLA (a PEGylated poly copolymer) named NP-PTX-DOX by self-assembling micelles in water and effectively encapsulating PTX and DOX, simultaneously (21). In response to reduction and acid stimulation, the prepared NP-PTX-DOX was triggered for targeted release into the OS tissue region. In this study, flow cytometry analysis and confocal laser scanning microscopy confirmed that dual-drug NPs were effectively internalized by K7 OS cells. The results showed that NP-PTX-DOX had a synergistic therapeutic effect and could induce apoptosis in K7 OS cells, exhibited a more optimized biological distribution, and had a higher tumor inhibition effect.

Curcumin (CUR), a potential antitumor drug, is limited by its low water solubility and low bioavailability. For the treatment of OS, a high affinity for bone tissue is the primary requirement for bone-targeted drug delivery. In bone tissue with tumor metastasis, the major component of skeletal hydroxyapatite is exposed to the lysis component. As a result, a higher affinity for hydroxyapatite indicates a more efficient bone tissue-targeting drug delivery capability. Xi et al. prepared alendronate hyaluronic acid octadecanoic acid (ALN-HA-C18), which is a stable spherical micelle nano-aggregate, loaded with CUR, with a uniform size and core-shell structure (22). ALN-HA-C18 nanomicelles and dual-targeted drug delivery systems with moderate particle sizes, high drug loading ability, remarkable slow-release properties, and hydroxyapatite, combined with the characteristics of strong affinity, can achieve effective drug delivery. Compared with free CUR, this drug system leads to a higher drug concentration in blood and tumor tissue, reduces drug distribution in the heart, and can send more CUR to OS tissues. Furthermore, it exhibited a higher cytotoxic activity against MG-63 cells, greatly improving *in vivo* antitumor activity, and reducing systemic toxicity.

In the absence of certain temperature conditions and ionic strength, nanomicelles may not remain stable (e.g., in solutions with ionic strength >200 mm and under low pH conditions) (81). Although nanomicelles with hydrophobic cores provide a superior carrier environment for the encapsulation of insoluble anticancer drugs, researchers still face challenges such as uncontrolled (non-continuous) drug release, immunogenicity, interactions with other soluble liquid molecules, and poor stability *in vivo*.

### 2.5 Dendrimer

Dendrimer NPs are three-dimensional polymers composed of a central nuclear atom, two repeating branch units, and end groups that affect molecular functions (82). The high ratio of end-base to molecular volume makes it a promising drug delivery vector. Drugs and oligonucleotides can be encapsulated in the internal cavities through hydrophobic and electrostatic interactions. The dendritic formulation contributes to the stability of its core components and provides a dynamic internal cavity where neutral molecules and ions can be loaded. Dendrimers also aid in site-specific targeted drug delivery through the conjugation of targeted ligands and dendrimer surfaces. Because dendritic molecules are uniform in size, they can easily cross cell membranes, so they help maintain the pharmacological activity of various drugs and improve their bioavailability (83). These nanostructures are well known for their special structure, excellent water solubility, high biocompatibility, and high encapsulation ability for a variety of molecules. These properties of dendritic macromolecules make them desirable vectors for drug delivery systems.

Although hydrogels have remarkable biocompatibility and can simulate biological tissues, their instability and uncontrolled release limit their applications in the biomedical field. Goncalves et al. reported a method for constructing stable AG/G5 NPs (23). Using CaCl2 as a crosslinker and amino terminally grafted 5-dendrimer (G5) as a crosslinker, they prepared a double-crosslinked dendrimer/alginate nanogel (AG/G5) by the emulsion method. G5 dendrimers can aid in the formation of AG/G5 NPs through strong electrostatic interactions with anionic alginates. The presence of G5 dendrimers can reduce the dosage of DOX by three times and release Dox slowly. The AG/G5 nanogel-loaded Dox was effectively absorbed by CAL-72 cells (human OS cell line) and delivered Dox in a targeted manner to perform its anticancer cell function. In particular, G5 dendritic macromolecules labeled with fluorescent molecules can be integrated into the nanogel, and once they enter the body, they can track the cancer cells using a fluorescence microscope. These results suggest that AG/G5 NP can be used as a promising drug delivery platform and biomonitor for OS.

Dendrimers have been used in drug delivery systems since the end of the 1990s owing to their unique advantages. However, certain studies have shown that dendrimers have certain drawbacks. Almost all types of dendrimers are cytotoxic and hemolytic, especially cations and high-generation dendrimers, and their toxicity is mainly related to the surface end groups (84). In addition, PAMAM dendrimers have been shown to have toxic effects on blood brain barrier cells, increasing membrane permeability and cellular calcium concentration in hippocampal neurons, thereby interfering with synaptic signals and causing neurotoxicity (85). Other studies have shown that the yield of high-generation dendrimers is low because of the stereoscopic obstacles encountered when branching to the core (86). However, scientists have found that surface modification of dendrimers can reduce their cytotoxicity problems, for example, by conjugation of molecules with the reactive terminal groups on these nanocarriers and specific elements (core, branch, and surface groups) that regulate dendrimers. Further study on dendrimers is required.

### 2.6 Nanocapsule

Nanocapsules are sub-micron-sized hollow NPs with unique nanostructures, consisting of a polymer shell of liquid and solid core (87). Because of their hollow core, nanocapsules can encapsulate drugs that are larger than traditional nanostructures, and effectively protect the pharmacological activities of these drugs. They have an unusually high specific surface area, which facilitates drug surface loading and interactions between ligand-modified nanocapsule drug delivery platforms, cell surface receptors, and other signaling molecules to achieve targeted drug delivery. They can be designed in a specific shape and size and can maintain the integrity of the structure in the process of circulating and against many unstable factors including blood flow in the shear stress and the interaction of serum albumin and dilution. Nanocapsules are becoming a research hotspot for drug delivery, gene delivery, drug-gene co-delivery, and other therapeutic drug delivery systems as well as biological imaging diagnostic applications owing to their numerous advantages.

Ifosfamide (IFS) is a DNA alkylating agent, which is a widely used antitumor drug that can inhibit DNA replication in OS cells and lead to OS cell apoptosis (88). Despite its superior antitumor effect, its clinical use is compromised by its free or natural form of toxicity. Wang et al. developed ifosfamide-loaded-lipid-core nanocapsules (IFS-LNCs), which have a narrow particle size distribution and a high drug-loading capacity (24). After entering the body of the OS model rats, IFS-LNCs were ingested by MG63 cancer cells through endocytosis, which may lead to accelerated destruction of acidic endososome vesicles. Subsequently, IFS was rapidly released into the cytoplasm and increased the apoptosis of OS cells by increasing the expression levels of Caspase-3 and Caspase-9 in MG63 cells. Overall, IFS-LNCs improved the therapeutic effects of OS.

Antisense oligonucleotides(AON) can be used to inhibit the EWS-FLI-1 oncogene (a pathogenic gene of Ewing sarcoma) (89). Nevertheless, due to their shortcomings such as easy degradation, short biological life span, and limited cell uptake, they have not fully played their role as clinical drugs. Based on this, Maksimenko et al. prepared nanocapsules by an interfacial polymerization method and combined AON with nanocapsules for the treatment of an OS rat model (25). CTAB, a cationic surfactant of AON-loaded NPs, can penetrate the endosomal membrane of the nucleus of OS, thereby inducing more efficient cytoplasmic delivery of AON. AON-loaded NPs can promote the mRNA specific downregulation of EWS-FLI -1 in the animal model of OS, thus effectively inhibiting the formation of OS from the source.

Many parameters determine the characteristics of the nanocapsules used in drug carriers, such as the capsule radius distribution of the surface, thickness, capsule, capsule membrane permeability, thermal decomposition, chemical decomposition, etc. As a result, whether nanocapsules can effectively target drug delivery drugs to tumor tissues *in vivo* depends on their strict preparation and preservation conditions, which make the promotion of nanocapsules in clinical applications a challenge.

### 2.7 Exosomes

Exosomes are natural extracellular, membranous, double-layer lipid membrane extracellular vesicles with a size of 30–100 nm secreted by living cells (90). They originate from intracellular multivesicles and are released into the extracellular microenvironment through exocytosis. Exosomes usually carry various genetic messenger bodies, such as DNA, mRNA, microRNA, cytoplasmic protein, and lipids, and transfer these regulatory molecules and genetic information from donor cells to recipient cells; therefore, they play a vital role in tumor proliferation, differentiation, metastasis, and resistance to chemotherapy or radiation (91). Tumor-derived exosomes have drawn the attention of scientists because of their specific biological activities. Exosomes are becoming an ideal drug delivery carrier, biometric marker, and liquid biopsy tool owing to their wide distribution in biological liquids, nanoscale dimensions, the ability to accurately position *in vivo*, and penetrate the blood-brain barrier.

Exosomes are secreted by many cell types, such as epithelial cells, hematopoietic cells, tumor cells, and mesenchymal stem cells (MSCs). Among these cells, MSCs are considered ideal candidates for the mass production of exosomes. Wei et al. prepared exosome-loaded DOX, named EXO-DOX, by isolating exosomes from bone marrow MSCs using an exosome separation kit, mixed them with DOX, desalted them with triethylamine, and finally treated them with PBS overnight; they were used for the treatment of OS (26). Acidic environments, such as advanced endosomes and lysosomes in OS cells can accelerate DOX release from EXO-DOX and make EXO-DOX more efficient than free DOX. As the main side effect of DOX is cardiotoxicity, the uptake rate of DOX by cardiac H9C2 cells was observed using inverted fluorescence microscopy and flow cytometry. The results showed that lower amounts of EXO-DOX entered cardiac H9C2 compared to free DOX, so the cytotoxicity of EXO-DOX to cardiac cells was lower than that of free DOX, which may be related to the interaction between MSC-derived exobody surface membrane proteins and MG63 cells. After cell viability analysis, EXO-DOX proved to have more optimized cytocompatibility. In conclusion, EXO-DOX has more optimized cell uptake, lower cardiotoxicity, and antitumor effects against OS than free DOX.

Exosomes can carry endogenous bioactive cargos, including proteins, DNA, and RNA (90). Gong et al. found that OS exosomes have high expression of miR675 but not cellular miR-675, and the exosome mir675 was found to be closely related to the metastatic phenotype in OS patients (92). In other words, if the expression of mir-675 in exosomes is reduced by chemical transfection, the metastasis of OS can be inhibited. Another study showed that BMSC-derived exosomes promote the expression of ERG by transporting lncRNA PVT1 to OS cells *via* miR-183-5p competitively binding and inhibiting the ubiquitination of ERG, thereby increasing the expression of ERG to promote osteosarcoma cell growth and transfer (93). The above research provides us with more information on the exosomal-based delivery in siRNA and miRNA therapeutics.

The potential use of exosomes for OS therapy was also demonstrated in a recent study by Sha et al., who found that the Wnt/β-catenin signaling pathway was upregulated in 50% of human sarcoma tissues and 65% of sarcoma cell lines. Activation of the TCF/β-catenin target gene promotes the proliferation of multiple sarcoma subtypes High expression of CDC25A, β-catenin, and some target genes may promote the occurrence and metastasis of OS, especially in lung metastasis. The HIC-5 gene was observed to interact with SMAD4 *in vivo* and upregulates Wnt/β-catenin signaling by reducing TCF/LEF activity, thus promoting the occurrence and development of OS (27). Based on these findings, Sha et al. proposed the use of exosomes to silence HIC-5 to prevent the occurrence of OS. They prepared exosome-mediated HIC-5 (HIC-5-EXO), which can effectively inhibit the expression of HIC-5 gene in MG-63 cells, thereby inactivating Wnt/β-catenin signaling and inhibiting OS cells and their metastasis (27). This study confirmed that HIC-5-EXO can effectively inhibit the proliferation of OS by inducing the apoptosis of target cells at the gene level, thus exerting its therapeutic effect on OS. Although exosomes are expected to be a promising tool for the detection and treatment of OS, they exhibit certain limitations. Currently, there may be a size difference between exosomes obtained *in vitro* and those secreted *in vivo* by patients; therefore, a new and noninvasive approach for obtaining tumor exosomes *in vivo* (such as from vaginal secretions, stool, or saliva) should be developed. In addition, the storage conditions of exosomes are extremely demanding, and the commonly used storage conditions are phosphate buffers at -80°C. Furthermore, studies have shown that low temperature can affect the stability of exosomes and result in the loss of content and corresponding biological functions (94). Thus, more mild and accessible culture conditions for exosomes should be studied.

## 3 Challenges and Future Perspective

Conventional surgery combined with chemotherapy remains the main treatment strategy for OS, but without sufficient promise and the possibility of tumor reappearance. Considering the other various shortcomings of this therapeutic approach, there is an urgent need to find NP-based drug delivery systems that deliver drugs specifically to the cancer site. These NP-based drug delivery systems can overcome many limitations of chemotherapy drugs, such as early blood clearance, acquired drug resistance, and toxic side effects, and provide novel treatment strategies for OS.

Over the past three decades, anti-cancer drug nanocarriers have achieved remarkable progress, and thousands of NPs have been modified and synthesized for drug delivery (95). However, not every kind of NP is suitable for loading anti-cancer drugs and for the treatment of every kind of tumor. When choosing the ideal anti-OS drug nanocarrier, it is necessary to consider the chemical properties, such as surface charge, modification, particle size, and hydrophobicity; pharmacological characteristics, such as drug binding rate, encapsulation rate, controlled release, reduced toxicity, and altered drug dissolution properties; anti-cancer properties, such as targeted delivery, photochemistry, photothermal, and photodynamic ability, as well as the biological characteristics, such as tumor tissue absorption, penetration of OS cells, and bioavailability (96, 97). Recent research has focused on hybrid formulations of liposomes or polymer shell inorganic NPs. These mixed carriers can effectively solve some of the limitations of liposomes, such as premature drug release, short storage time, burst release, leaching, poor particle size control, and insufficient drug loading, and can also address the toxicity issue of inorganic nanomaterials. For example, as one of the common post-surgery chemotherapy medicines, DOX often causes toxic side effects in normal tissues and organs, including inhibition of bone marrow hematopoietic function, such as thrombocytopenia and leukopenia. It can induce cardiotoxicity and even heart failure, and cause fever, hemorrhagic erythema, and liver damage. Recently, an *in vitro* study indicated that lipid-based nanocarriers successfully encapsulating DOX improved cellular uptake of DOX, bypassing cell resistance mediated by p-glycoprotein 1 in targeted OS cells (98). On the other hand, DOX and free liposomes showed a synergistic effect in cell experiments, which shows that lipid-based NPs combined with these anti-tumor drugs can reduce the drug dose required by an individual, thereby ensuring a more effective treatment. The results showed that drug-loaded nanogels released about 38.5% of the drug under the physiological environment of pH=7 and 86% under the acidic tumor environment of pH=5.5, proving that NPs have pH-selective release characteristics and the ability to target OS tumors (98).

In addition, most NP synthesis formulas contain toxic reagents; for example, the synthesis of PLGA requires the use of the organic reagent acetone, whereas the synthesis of polydopamine requires the use of ethanol in the process of coating metal nano cores (99, 100). It is worth mentioning that many NP synthesis formulas contain more than one toxic reagent, and these highly toxic substances are difficult to remove through chemical modification or repeated washing. Therefore, the standard for evaluating the toxicity of nanoparticle carriers is *in vivo* and *in vitro* toxicity experiments, such as hemolysis experiments, macrophage and normal cell killing experiments, normal tissue release experiments, and toxicity evaluation in animal models (101). More importantly, long-term pre-clinical evaluation of Fe_3_O_4_, Ag, and Au NPs in the body takes up to a decade to several decades of metabolism (102).

For a quick transition of nanomaterials to industrial application, researchers need to understand and control complex factors that affect the pharmacology, safety, and efficacy of drug delivery systems to obtain controllable nanomaterials. At present, more than ten kinds of carrier-mediated drugs for the treatment of tumors, such as liposomal doxorubicin, pegfilgrastim, and belantamab mafodotin, have been approved by the US Food and Drug Administration (103). The biggest challenge in the clinical translation of NPs as nanocarriers is how to control their distribution and removal *in vivo* (103). Furthermore, a large number of studies on polymer nanocarriers are mainly focused on elucidating their mechanism of action, environmental response, and active targeting, and there is a lack of *in vivo* safety evaluation and phase III clinical trial results. The clinical translation of polymers mainly addresses issues such as their interaction with cell membranes and resulting increased toxicity, low drug retention and rapid clearance from blood circulation

There are still many challenges that need to be overcome in the translation of nanomaterials from research laboratories to clinical implementations, such as (1) nanoparticle toxicity and biological safety (2), high immune clearance rate, which affects targeting, and (3) lack of valid *in vitro* and *in vivo* experimental data for animal models and clinical patients. Surface functional modification, such as surface protein increase, pegylation, and charge modification, can be used to address the issue of the rapid clearance of NPs in the extremely complex immune system. Further, long-term *in vitro* and *in vivo* studies should be conducted to evaluate the toxicity and biological safety of NPs. Moreover, animal models related to clinical applications other than small animal models should be designed to verify the efficacy of nano-drug-loaded particles to improve the clinical translation of drug-delivery NPs.

Significant improvement is required to better optimize the applications of NPs in the clinical treatment of OS. First, reducing the cytotoxic immune response and prolonging blood circulation through surface modification of cell membranes or bionic proteins is required. Second, optimization of the size of nanoplatforms is necessary to improve the stability of NPs *in vivo*. In addition, the design of rational clinical trials and *in vivo* experiments is crucial. These problems seem to be addressable in future experiments, but further explorations are required in the field of OS treatment.

## 4 Conclusions

Although various chemotherapy approaches have been developed for OS patients to improve survival and quality of life, the prognosis of patients with late stage OS or metastatic OS still remains poor, and patients suffer from unbearable pain. Many tumor complications, such as distant metastasis, drug resistance, and cachexia, cannot be overcome by a single chemotherapy drug. Therefore, it is urgent that researchers find alternative therapeutic NP-based drug delivery systems to more effectively eliminate OS tumors.

As with other OS therapies, nanoplatform-based drug delivery is an emerging research field aimed at reducing the degradation of the drug in the blood and accurately delivering it to the tumor site. The use of nanoplatform-based therapy has many advantages compared with the existing methods for OS treatment, such as EPR effect, passive or active drug delivery, pH sensitivity, and NP surface modification (104). For example, liposomes are very similar to cell membranes and serve as a promising drug carrier that can simultaneously load hydrophobic and hydrophilic drugs and can also be modified for targeted or passive stimuli-responsive therapy. Compared to other synthetic nanomaterials, liposomes have superior biocompatibility and nontoxicity, and have even been used in clinical practice for the treatment of OS. However, some problems still need to be solved, such as drug leakage, aggregation, and inefficient encapsulation (105). Silica NPs can be used as stimuli-responsive drug delivery systems to efficiently encapsulate chemotherapy drugs; moreover, the mesoporous forms effectively increase the drug loading ratio in treating OS. Therefore, it is worth noting that concentrations must be used within safe ranges to avoid bio-incompatibility (6). Nano-oxides have great potential owing to their targeted drug delivery, delivering local hyperthermia along with a magneto mechanical effect on cancerous cells. Nano zirconia, nano cerium oxide, and nano iron oxide NPs are widely studied materials and are at the forefront of the theragnostic arena. Exosomes are a promising drug delivery tool because they are generally safe, targeted to tissues, and have excellent cellular uptake efficiency. Advanced bioengineering technology could improve the drug loading efficiency and targeting capabilities of exosomes, but long-term stability is a challenge of cell exosome-derived nanomaterials for clinical translation. Furthermore, exosome delivery systems are an emerging field; the major obstacle is low yields (105). Development of an optimal workflow and exploration of new methods over the new few years could overcome this problem. We hope that nanoplatform therapy strategies will be further optimized and developed to meet clinical treatment applications, thereby contributing remarkably to the treatment of OS patients.

## Author Contributions

KW collected, analyzed the data, and drafted the manuscript. BY, DL and YT participated in the data analysis and interpretation. JJ and YL revised and edited the final version of the manuscript. All authors read and approved the final manuscript.

## Funding

This study was financially supported by the Science and Technology Development Plan Projects of Jilin Province (Grant No. 20200201558JC), the Bethune project of Jilin University (Grant No. 2018A03).

## Conflict of Interest

The authors declare that the research was conducted in the absence of any commercial or financial relationships that could be construed as a potential conflict of interest.

## Publisher’s Note

All claims expressed in this article are solely those of the authors and do not necessarily represent those of their affiliated organizations, or those of the publisher, the editors and the reviewers. Any product that may be evaluated in this article, or claim that may be made by its manufacturer, is not guaranteed or endorsed by the publisher.
